# Retraction: Differential temporal inhibition of mitochondrial fission by Mdivi-1 exerts effective cardioprotection in cardiac ischemia/reperfusion injury

**DOI:** 10.1042/CS20180510_RET

**Published:** 2025-06-23

This article “Differential temporal inhibition of mitochondrial fission by Mdivi-1 exerts effective cardioprotection in cardiac ischemia/reperfusion injury” DOI: 10.1042/CS20180510 Maneechote et al., Clin Sci (Lond) (2018) 132 (15): 1669–1683 is being retracted from *Clinical Science* at the request of the Editor-in-Chief and the Editorial Board. It has been noted that multiple protein bands in Figure 5C Cyt c panel and Figure 5D Cleaved caspase-3 panel appear similar with rearrangement. Additionally, the Western blots in Figure 3B, Figures 5A and C, and Figures 8A and B appears to contain signs of digital splicing.

The authors were contacted regarding the concerns and stated that the Total Cx43 bands came from three separate blots and were spliced into Figures 3B. The other splice sites were the result of the ‘sham’ group being added separately to the figures as this condition was not included in the original experiments. They also provided a corrected version of Figure 5 and original data for the Western blots; however, this has not fully resolved the issues identified. The journal no longer has confidence in the reliability or validity of the results presented in this paper, and given the extent of the issues raised, the Editorial Board stands by the decision to retract the article. The authors disagree to the retraction.

